# The synthesis, crystal structure and spectroscopic analysis of (*E*)-3-(4-chloro­phen­yl)-1-(2,3-di­hydro­benzo[*b*][1,4]dioxin-6-yl)prop-2-en-1-one

**DOI:** 10.1107/S2056989023005613

**Published:** 2023-06-30

**Authors:** Vinaya, Austin S. Richard, Subbiah M. Murthy, Yeriyur B. Basavaraju, Hemmige S. Yathirajan, Sean Parkin

**Affiliations:** a Department of Studies in Chemistry, University of Mysore, Manasagangotri, Mysuru 570 006, India; b Aavishkaar Research Centre, Coorg Institute of Dental Sciences, Virajpet 571 218, India; c Department of Microbiology, Yuvaraja’s College, Mysore 570 005, India; d Department of Chemistry, University of Kentucky, Lexington, KY 40506-0055, USA; Katholieke Universiteit Leuven, Belgium

**Keywords:** crystal structure, chalcone, π–π inter­action

## Abstract

The synthesis, crystal structure and spectroscopic analysis of (*E*)-1-(2,3-di­hydro­benzo[*b*][1,4]dioxin-6-yl)-3-(4-chloro­phen­yl)prop-2-en-1-one (C_17_H_13_ClO_3_), a substituted chalcone, are described. The overall geometry of the mol­ecule is largely planar (r.m.s. deviation = 0.1742 Å), but slightly kinked, leading to a dihedral angle between the planes of the benzene rings at either side of the mol­ecule of 8.31 (9)°. In the crystal, only weak inter­actions determine the packing motifs. These include C—H⋯O and C—H⋯Cl hydrogen bonds and π–π overlap of aromatic rings.

## Abstract 2

The synthesis, crystal structure and some spectroscopic details for (*E*)-1-(2,3-di­hydro­benzo[*b*][1,4]dioxin-6-yl)-3-(4-chloro­phen­yl)prop-2-en-1-one are presented.

## 1. Chemical context

Chalcones exhibit a wide range of fascinating biological and pharmacological properties. Some of their beneficial attributes include anti-inflammatory, anti­microbial, anti­fungal, anti­oxidant, cytotoxic and anti­cancer activities (Dimmock *et al.*, 1999 ▸). Moreover, several chalcones have demonstrated significant anti­malarial properties (Troeberg *et al.*, 2000 ▸). The efficient second-harmonic generation (SHG) conversion efficiency of some chalcones has also made them promising organic nonlinear optical (NLO) materials (Sarojini *et al.*, 2006 ▸). The presence of phenolic groups in many chalcones has garnered attention due to their radical quenching capabilities, making these compounds and chalcone-rich plant extracts potential candidates for drug development or food preservation (Dhar *et al.*, 1981 ▸; Di Carlo *et al.*, 1999 ▸). The design, synthesis and evaluation of 1,4-benzodioxane-substituted chalcones as selective and reversible inhibitors of human mono­amine oxidase B was reported by Kong *et al.* (2020 ▸). Furthermore, Shinde *et al.* (2020 ▸) have conducted experimental and theoretical studies on the mol­ecular structure, FT–IR, NMR, HOMO/LUMO frontier orbital, mol­ecular electrostatic potential (MESP) and reactivity descriptors of (*E*)-1-(2,3-di­hydro­benzo[*b*][1,4]dioxin-6-yl)-3-(3,4,5-tri­meth­oxy­phen­yl)prop-2-en-1-one. They have also reported the synthesis, anti­bacterial and computational studies of halo-chalcone hybrids derived from 1-(2,3-di­hydro­benzo[*b*][1,4]dioxin-6-yl)ethan-1-one (Shinde *et al.*, 2021 ▸) and of two trifluorinated chalcones derived from 1-(2,3-di­hydro­benzo[*b*][1,4]dioxin-6-yl)ethan-1-one (Shinde *et al.*, 2022*a* ▸), as well as investigations of (*E*)-4-(3-(2,3-di­hydro­benzo[*b*][1,4]dioxin-6-yl)-3-oxoprop-1-en-1-yl)ben­zo­nitrile (Shinde *et al.*, 2022*b* ▸). Additionally, Zhuang *et al.* (2017 ▸) presented a comprehensive review on the chalcone framework as a privileged structure in medicinal chemistry, while Elkanzi *et al.* (2022 ▸) have published a review on the synthesis of chalcone derivatives and their biological activities. 
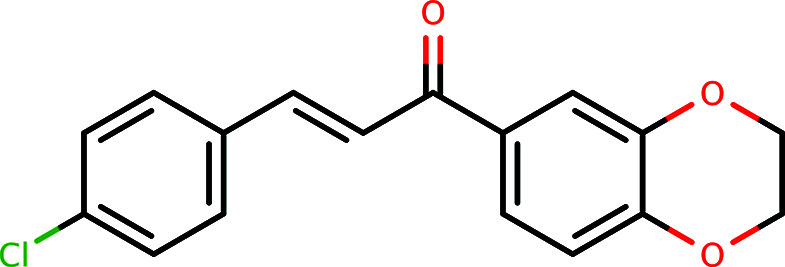


Given the general significance of chalcones, we report herein the synthesis of (*E*)-1-(2,3-di­hydro­benzo[*b*][1,4]dioxin-6-yl)-3-(4-chloro­phen­yl)prop-2-en-1-one, C_17_H_13_ClO_3_ (**I**), along with its crystal structure and related studies.

## 2. Structural commentary

**Figure 1:**
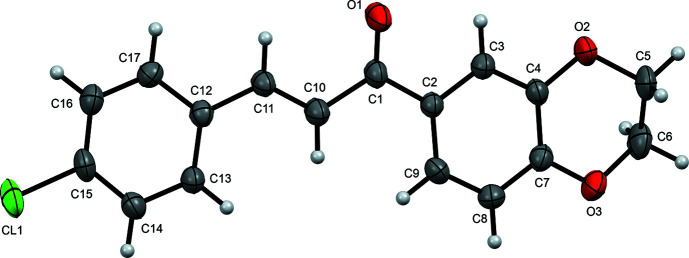
The mol­ecular structure of the title compound, showing the atom labelling and displacement ellipsoids drawn at the 50% probability level.

As shown in Fig. 1 ▸, the overall conformation of the mol­ecule of **I** is determined by the torsion angles C3—C2—C1—C10 [169.9 (2)°], C2—C1—C10—C11 [−171.3 (3)°], C1—C10—C11—C12 [−179.0 (3)°] and C10—C11—C12—C17 [−176.7 (3)°] of the propenone moiety. In magnitude, these are all within about 10° of 180°, which makes the mol­ecule largely flat (r.m.s. deviation for the non-H atoms is 0.1742 Å), though slightly bent, leading to a dihedral angle between the chloro­phenyl and di­hydro­benzodioxine rings of 8.31 (9)°. The conformation of the di­hydro­dioxine ring, as determined by its Cremer–Pople puckering parameters (Cremer & Pople, 1975 ▸) [*Q* = 0.466 (3) Å, θ = 51.2 (4)° and φ = 282.6 (4)°], is closest to half-chair (Boeyens, 1978 ▸). This puckering places atoms C5 and C6 at 0.499 (4) and −0.207 (4) Å on either side of the mean plane of the di­hydro­benzodioxine ring. All bond lengths and angles lie within the typical ranges found in organic structures.

## 3. Supra­molecular features

**Figure 2:**
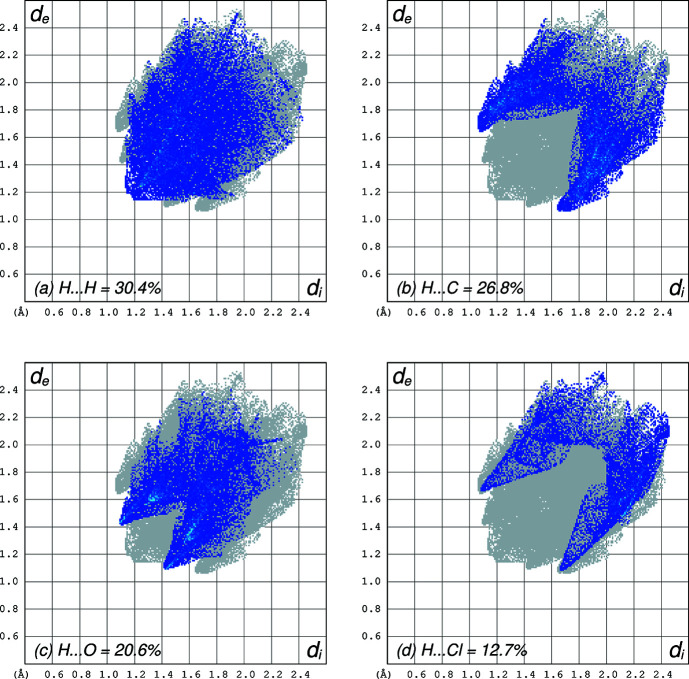
Hirshfeld surface fingerprint plots showing (*a*) H⋯H contacts = 30.4% coverage, (*b*) H⋯C = 26.8%, (*c*) H⋯O = 20.6% and (*d*) H⋯Cl = 12.7%. Reciprocal contacts are included in the coverage fractions. Other contact types were unremarkable.

**Figure 3:**
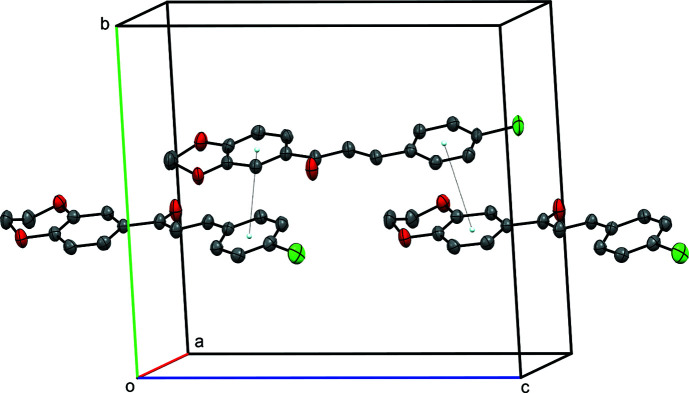
A partial packing plot of **I**, viewed approximately down the *a* axis. The stacking of the aromatic rings is shown by dotted lines between ring centroids, which lead to chains of mol­ecules that extend parallel to [001].

The crystal packing in **I** is governed solely by weak inter­actions, as the only possible hydrogen-bond donors are of type C—H. The absence of any sharp spikes protruding to the lower left in the Hirshfeld surface fingerprint plots (Fig. 2 ▸) calculated using *CrystalExplorer* (Spackman *et al.*, 2021 ▸) clearly marks the absence of any very close contacts. Indeed, only two such contacts are flagged as inter­molecular hydrogen-bond-type inter­actions by the default *SHELXL* HTAB command. These are C5—H5*A*⋯O3^i^ and C5—H5*B*⋯Cl^ii^ [symmetry codes: (i) *x* − 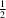 , −*y* + 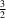 , *z*; (ii) *x* − 1, *y*, *z* − 1], shown in Table 1 ▸. The benzene rings of the 4-chloro­phenyl and di­hydro­benzodioxine groups, however, exhibit π–π overlap, which link the mol­ecules into chains that extend parallel to [001], as shown in Fig. 3 ▸. The centroid–centroid distances [3.747 (18) Å] and the dihedral angle between the participating rings [6.12 (11)°] imply that these are rather weak inter­actions.

## 4. Database survey

A search of the Cambridge Structural Database (CSD, Version 5.43 with updates to November 2022; Groom *et al.*, 2016 ▸) using a mol­ecular fragment consisting of chalcone (C_6_H_5_C=OCH=CHC_6_H_5_) with no specific substituents returned 4725 hits. With hydrogen attached across the C=C double bond, the search gave 1200 hits. Of these entries, 155 had ‘any halogen’ at the equivalent of C15 in **I**, 62 of which had Cl. A search fragment without the halogen but with ‘any oxygen bound’ substituent at the equivalent of C4 and C7 pro­duced 78 matches. Addition of the aforementioned halogen reduced this to 14 structures, seven of which were chlorides, but only five are unique. These are: BOJFIQ [(2*E*)-1-(1,3-benzodioxol-5-yl)-3-(4-chloro­phen­yl)prop-2-en-1-one; Jasinski *et al.*, 2008 ▸], FATFIR [(*E*)-3-(2,4-di­chloro­phen­yl)-1-(3,4,5-tri­meth­oxy­phen­yl)prop-2-en-1-one; Wu *et al.*, 2012 ▸], TICDIT [3-(4-chloro­phen­yl)-1-(3,4-di­meth­oxy­phen­yl)prop-2-en-1-one; Teh *et al.*, 2007 ▸], VIDDIW [3-(2,4-di­chloro­phen­yl)-1-(3,4-di­meth­oxy­phen­yl)prop-2-en-1-one; Ng *et al.*, 2007 ▸] and XOLLOC [3-(4-chloro-3-fluoro­phen­yl)-1-(3,4-di­meth­oxy­phen­yl)prop-2-en-1-one; Çelikesir *et al.*, 2019 ▸]. A few other related chalcone structures include: QERYOC [(2*E*)-3-(1,3-benzodioxol-5-yl)-1-(4-bromo­phen­yl)prop-2-en-1-one; Harrison *et al.*, 2006 ▸], KUYWOR [(2*E*)-3-(1,3-ben­zodioxol-5-yl)-1-(3-bromo-2-thien­yl)prop-2-en-1-one; Har­rison *et al.*, 2010 ▸], TUNTAY [(2*E*)-1-(1,3-benzodioxol-5-yl)-3-(2-bromo­phen­yl)prop-2-en-1-one; Li *et al.*, 2010 ▸], and UNUZUZ {(*E*)-3-(8-ben­z­yloxy-2,3-di­hydro-1,4-benzodioxin-6-yl)-1-[2-hy­droxy-4,6-bis­(meth­oxy­meth­oxy)phen­yl]prop-2-en-1-one; Zhang *et al.*, 2011 ▸}.

## 5. Synthesis, crystallization, and spectroscopic data

**Figure 4:**
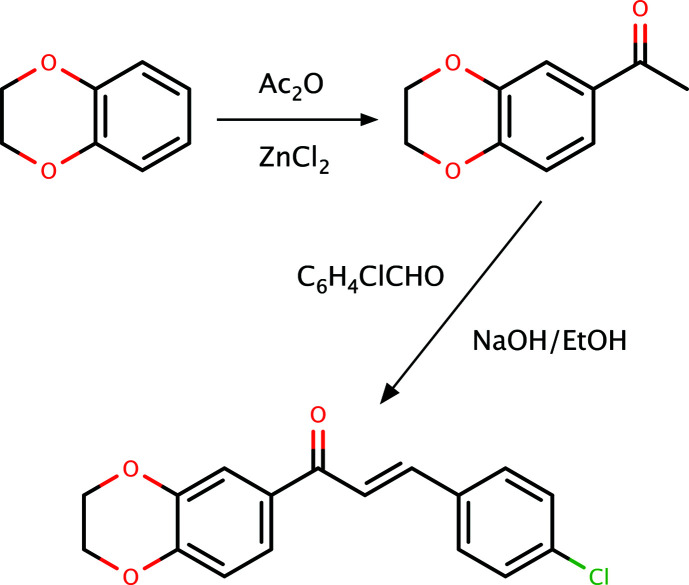
A generalized reaction scheme for the synthesis of **I**.

1-(2,3-Di­hydro­benzo[1,4]dioxin-6-yl)ethanone was prepared by Friedel–Crafts acyl­ation of benzo[1,4]dioxane using ether as solvent (Fig. 4 ▸). 1-(2,3-Di­hydro­benzo[1,4]dioxin-6-yl)ethanone (1.78 g, 0.01 mol) and 4-chloro­benzaldehyde (1.40 g, 0.01 mol) were stirred in a 30% ethano­lic NaOH and water mixture at 293 K for 4–6 h. The reaction mixture was refrigerated overnight. The precipitate that formed was filtered off and dried. X-ray-quality crystals were obtained from a solvent mixture of DMF–DMSO (di­methyl­formamide–dimethyl sulfoxide) (1:1 *v*/*v*) and the corresponding melting point was found to be 410–411 K.

FT–IR (ν in cm^−1^): 3156–2934 (Ar-CH), 1655 (C=O), 1575 (C=C); ^1^H NMR (CDCl_3_, 400 MHz): δ 7.71 (*d*, 1H, β-CH), 7.92 (*d*, 2H, Ar-H), 7.62 (*d*, 2H, Ar-H), 7.23 (*d*, 1H, 2,3-di­hydro­benzo[1,4]dioxine Ar-H), 7.21 (*d*, 1H, 2,3-di­hydro­benzo[1,4]dioxine Ar-H), 7.01 (*d*, 1H, α-CH), 6.93 (*s*, 1H, 2,3-di­hydro­benzo[1,4]dioxine Ar-H), 4.14 (*m*, 4H, 1,4-dioxane CH_2_); ^13^C NMR (CDCl_3_, 100 MHz): δ 196.1, 156.5, 149.8, 145.1, 133.5, 133.3, 129.0, 128.7, 122.1, 121.4, 121.3, 112.0, 106.8, 64.2.

## 6. Refinement

Crystal data, data collection and structure refinement details are summarized in Table 2 ▸. All H atoms were found in difference Fourier maps and subsequently included in the refinement using riding models, with constrained distances set to 0.95 [C(*sp* ^2^)H] and 0.99 Å (*R*_2_CH_2_). *U*_iso_(H) values were set to 1.2*U*_eq_ of the attached atom. The absolute structure was determined using 1035 quotients of the form [(*I* ^+^) − (*I* ^−^)]/[(*I* ^+^) + (*I* ^−^)] (Parsons *et al.*, 2013 ▸).

## Supplementary Material

Crystal structure: contains datablock(s) I, global. DOI: 10.1107/S2056989023005613/vm2285sup1.cif

Structure factors: contains datablock(s) I. DOI: 10.1107/S2056989023005613/vm2285Isup2.hkl

CCDC reference: 2272334

Additional supporting information:  crystallographic information; 3D view; checkCIF report

## Figures and Tables

**Table 1:** Hydrogen-bond geometry (Å, °)

*D*—H⋯*A*	*D*—H	H⋯*A*	*D*⋯*A*	*D*—H⋯*A*
C5—H5*A*⋯O3^i^	0.99	2.61	3.595 (4)	172
C5—H5*B*⋯Cl1^ii^	0.99	2.80	3.455 (3)	124

Symmetry codes: (i) 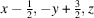 ; (ii) 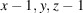 .

**Table 2:** Experimental details

Crystal data	
Chemical formula	C_17_H_13_ClO_3_
*M*_r_	300.72
Crystal system, space group	Orthorhombic, *P* *n* *a*2_1_
Temperature (K)	200
*a*, *b*, *c* (Å)	5.8655 (5), 14.3499 (17), 16.4803 (19)
*V* (Å^3^)	1387.1 (3)
*Z*	4
Radiation type	Mo *K*α
μ (mm^−1^)	0.28
Crystal size (mm)	0.26 × 0.23 × 0.22

Data collection	
Diffractometer	Bruker D8 Venture dual source
Absorption correction	Multi-scan (*SADABS*; Krause *et al.*, 2015 ▸)
*T*_min_, *T*_max_	0.788, 0.971
No. of measured, independent and observed [*I* > 2σ(*I*)] reflections	17057, 2896, 2694
*R*_int_	0.038
(sin θ/λ)_max_ (Å^−1^)	0.651

Refinement	
*R*[*F* ^2^ > 2σ(*F* ^2^)], *wR*(*F* ^2^), *S*	0.033, 0.083, 1.07
No. of reflections	2896
No. of parameters	190
No. of restraints	1
H-atom treatment	H-atom parameters constrained
Δρ_max_, Δρ_min_ (e Å^−3^)	0.21, −0.16
Absolute structure	Flack *x* determined using 1035 quotients [(*I* ^+^) − (*I* ^−^)]/[(*I* ^+^) + (*I* ^−^)] (Parsons *et al.*, 2013 ▸)
Absolute structure parameter	0.01 (2)

Computer programs: *APEX3* (Bruker, 2016 ▸), *SHELXT* (Sheldrick, 2015*a* ▸), *SHELXL2019* (Sheldrick, 2015*b* ▸), *Mercury* (Macrae *et al.*, 2020 ▸), *SHELX* (Sheldrick, 2008 ▸) and *publCIF* (Westrip, 2010 ▸).

## supplementary crystallographic information

### Crystal data

**Table:**

C_17_H_13_ClO_3_	*D*_x_ = 1.440 Mg m^−^^3^
*M**_r_* = 300.72	Mo *K*α radiation, λ = 0.71073 Å
Orthorhombic, *P**n**a*2_1_	Cell parameters from 9943 reflections
*a* = 5.8655 (5) Å	θ = 2.5–27.6°
*b* = 14.3499 (17) Å	µ = 0.28 mm^−^^1^
*c* = 16.4803 (19) Å	*T* = 200 K
*V* = 1387.1 (3) Å^3^	Cut block, pale yellow
*Z* = 4	0.26 × 0.23 × 0.22 mm
*F*(000) = 624	

### Data collection

**Table:**

Bruker D8 Venture dual source diffractometer	2896 independent reflections
Radiation source: microsource	2694 reflections with *I* > 2σ(*I*)
Detector resolution: 7.41 pixels mm^-1^	*R*_int_ = 0.038
φ and ω scans	θ_max_ = 27.6°, θ_min_ = 2.5°
Absorption correction: multi-scan (*SADABS*; Krause *et al.*, 2015)	*h* = −7→7
*T*_min_ = 0.788, *T*_max_ = 0.971	*k* = −18→18
17057 measured reflections	*l* = −19→21

### Refinement

**Table:**

Refinement on *F*^2^	Secondary atom site location: difference Fourier map
Least-squares matrix: full	Hydrogen site location: difference Fourier map
*R*[*F*^2^ > 2σ(*F*^2^)] = 0.033	H-atom parameters constrained
*wR*(*F*^2^) = 0.083	*w* = 1/[σ^2^(*F*_o_^2^) + (0.0322*P*)^2^ + 0.4666*P*] where *P* = (*F*_o_^2^ + 2*F*_c_^2^)/3
*S* = 1.07	(Δ/σ)_max_ < 0.001
2896 reflections	Δρ_max_ = 0.21 e Å^−^^3^
190 parameters	Δρ_min_ = −0.16 e Å^−^^3^
1 restraint	Absolute structure: Flack *x* determined using 1035 quotients [(I+)-(I-)]/[(I+)+(I-)] [Parsons *et al.* (2013)
Primary atom site location: structure-invariant direct methods	Absolute structure parameter: 0.01 (2)

### Special details

**Table:**

Experimental. The crystal was mounted using polyisobutene oil on the tip of a fine glass fibre, which was fastened in a copper mounting pin with electrical solder. It was placed directly into the cold gas stream of a liquid-nitrogen based cryostat (Hope, 1994; Parkin & Hope, 1998). The crystals appeared to undergo a destructive phase transition when cooled to 90K. Visual inspection of crystal integrity and diffraction quality vs temperature established a safe temperature for data collection of -73° C.
Geometry. All esds (except the esd in the dihedral angle between two l.s. planes) are estimated using the full covariance matrix. The cell esds are taken into account individually in the estimation of esds in distances, angles and torsion angles; correlations between esds in cell parameters are only used when they are defined by crystal symmetry. An approximate (isotropic) treatment of cell esds is used for estimating esds involving l.s. planes.
Refinement. Refinement progress was checked using *Platon* (Spek, 2020) and by an *R*-tensor (Parkin, 2000). The final model was further checked with the IUCr utility *checkCIF*.

### Fractional atomic coordinates and isotropic or equivalent isotropic displacement parameters (Å^2^)

**Table:**

	*x*	*y*	*z*	*U*_iso_*/*U*_eq_	
Cl1	0.86056 (16)	0.65710 (6)	0.91506 (5)	0.0577 (3)	
O1	−0.0096 (3)	0.59370 (18)	0.48876 (12)	0.0505 (6)	
O2	0.0030 (3)	0.57318 (14)	0.18914 (12)	0.0416 (5)	
O3	0.4399 (4)	0.64129 (16)	0.14256 (12)	0.0443 (5)	
C1	0.1903 (5)	0.6112 (2)	0.47247 (15)	0.0338 (6)	
C2	0.2634 (4)	0.61897 (18)	0.38646 (15)	0.0286 (5)	
C3	0.1080 (4)	0.59307 (18)	0.32641 (17)	0.0318 (5)	
H3	−0.038161	0.570520	0.341626	0.038*	
C4	0.1637 (4)	0.59976 (17)	0.24503 (16)	0.0300 (5)	
C5	0.0414 (6)	0.6143 (2)	0.11107 (17)	0.0453 (7)	
H5A	0.008461	0.681919	0.113519	0.054*	
H5B	−0.062554	0.585853	0.070731	0.054*	
C6	0.2829 (6)	0.5995 (2)	0.08557 (18)	0.0475 (8)	
H6A	0.313824	0.531780	0.081738	0.057*	
H6B	0.306758	0.627076	0.031178	0.057*	
C7	0.3778 (5)	0.63300 (19)	0.22209 (16)	0.0320 (6)	
C8	0.5313 (5)	0.66010 (18)	0.28102 (18)	0.0343 (6)	
H8	0.675979	0.683954	0.265581	0.041*	
C9	0.4761 (5)	0.65280 (18)	0.36243 (17)	0.0329 (6)	
H9	0.584046	0.671004	0.402365	0.040*	
C10	0.3628 (5)	0.62304 (19)	0.53720 (16)	0.0361 (6)	
H10	0.511960	0.643759	0.523877	0.043*	
C11	0.3090 (5)	0.60465 (18)	0.61436 (15)	0.0314 (6)	
H11	0.158246	0.583195	0.624038	0.038*	
C12	0.4563 (4)	0.61380 (17)	0.68570 (15)	0.0297 (5)	
C13	0.6738 (4)	0.65334 (18)	0.68259 (17)	0.0326 (5)	
H13	0.737075	0.671519	0.631943	0.039*	
C14	0.7979 (5)	0.66609 (18)	0.75398 (18)	0.0347 (6)	
H14	0.945533	0.693264	0.752293	0.042*	
C15	0.7047 (5)	0.63898 (18)	0.82670 (18)	0.0355 (6)	
C16	0.4927 (5)	0.59816 (19)	0.83158 (18)	0.0367 (6)	
H16	0.432553	0.578743	0.882366	0.044*	
C17	0.3696 (5)	0.58612 (18)	0.76102 (16)	0.0330 (6)	
H17	0.222733	0.558409	0.763644	0.040*	

### Atomic displacement parameters (Å^2^)

**Table:**

	*U*^11^	*U*^22^	*U*^33^	*U*^12^	*U*^13^	*U*^23^
Cl1	0.0663 (5)	0.0745 (5)	0.0324 (4)	−0.0046 (4)	−0.0248 (4)	0.0048 (4)
O1	0.0324 (10)	0.0939 (18)	0.0252 (10)	−0.0057 (11)	0.0000 (9)	0.0001 (10)
O2	0.0414 (11)	0.0586 (12)	0.0248 (9)	−0.0105 (10)	−0.0082 (9)	0.0018 (9)
O3	0.0440 (11)	0.0634 (14)	0.0254 (10)	−0.0025 (10)	0.0040 (9)	0.0059 (9)
C1	0.0345 (14)	0.0442 (15)	0.0226 (13)	0.0005 (12)	−0.0028 (11)	−0.0017 (12)
C2	0.0283 (12)	0.0340 (13)	0.0234 (12)	0.0011 (10)	−0.001 (1)	0.0006 (10)
C3	0.0312 (12)	0.0381 (13)	0.0261 (13)	−0.0009 (10)	−0.0011 (11)	0.0015 (11)
C4	0.0313 (13)	0.0327 (12)	0.0259 (12)	−0.0001 (11)	−0.0058 (11)	0.0007 (10)
C5	0.0526 (18)	0.0612 (18)	0.0221 (13)	−0.0025 (15)	−0.0101 (13)	0.0041 (13)
C6	0.0591 (19)	0.062 (2)	0.0217 (13)	0.0047 (16)	−0.0007 (14)	−0.0005 (13)
C7	0.0326 (14)	0.0374 (13)	0.0259 (13)	0.0032 (11)	0.0014 (11)	0.004 (1)
C8	0.0288 (13)	0.0410 (14)	0.0330 (14)	−0.0026 (11)	0.0014 (12)	0.0034 (12)
C9	0.0303 (12)	0.0380 (14)	0.0305 (13)	−0.0033 (10)	−0.0059 (11)	−0.0012 (11)
C10	0.0326 (13)	0.0489 (16)	0.0268 (14)	−0.0015 (13)	−0.0020 (11)	−0.0015 (13)
C11	0.0291 (13)	0.0403 (14)	0.0247 (13)	−0.0004 (11)	−0.0012 (10)	−0.0031 (11)
C12	0.0316 (13)	0.0330 (13)	0.0246 (13)	0.0023 (10)	0.0005 (11)	−0.0017 (11)
C13	0.0324 (13)	0.0396 (13)	0.0259 (13)	−0.0005 (10)	−0.0024 (11)	0.0010 (11)
C14	0.0321 (13)	0.0387 (14)	0.0332 (14)	0.0003 (11)	−0.0069 (12)	0.0010 (12)
C15	0.0416 (15)	0.0393 (13)	0.0255 (12)	0.0060 (11)	−0.0117 (12)	0.0000 (11)
C16	0.0430 (15)	0.0416 (14)	0.0256 (13)	0.0023 (12)	−0.0005 (12)	0.0033 (12)
C17	0.0337 (13)	0.0362 (13)	0.0292 (14)	−0.0005 (10)	0.0021 (11)	0.0013 (11)

### Geometric parameters (Å, º)

**Table:**

Cl1—C15	1.739 (3)	C7—C8	1.380 (4)
O1—C1	1.229 (3)	C8—C9	1.384 (4)
O2—C4	1.372 (3)	C8—H8	0.9500
O2—C5	1.433 (3)	C9—H9	0.9500
O3—C7	1.366 (3)	C10—C11	1.336 (4)
O3—C6	1.446 (4)	C10—H10	0.9500
C1—C10	1.480 (4)	C11—C12	1.465 (4)
C1—C2	1.485 (3)	C11—H11	0.9500
C2—C3	1.396 (4)	C12—C13	1.397 (4)
C2—C9	1.396 (4)	C12—C17	1.399 (4)
C3—C4	1.384 (4)	C13—C14	1.396 (4)
C3—H3	0.9500	C13—H13	0.9500
C4—C7	1.395 (4)	C14—C15	1.373 (4)
C5—C6	1.493 (5)	C14—H14	0.9500
C5—H5A	0.9900	C15—C16	1.377 (4)
C5—H5B	0.9900	C16—C17	1.379 (4)
C6—H6A	0.9900	C16—H16	0.9500
C6—H6B	0.9900	C17—H17	0.9500
			
C4—O2—C5	112.4 (2)	C7—C8—H8	119.7
C7—O3—C6	114.7 (2)	C9—C8—H8	119.7
O1—C1—C10	121.2 (2)	C8—C9—C2	120.7 (2)
O1—C1—C2	120.0 (2)	C8—C9—H9	119.7
C10—C1—C2	118.8 (2)	C2—C9—H9	119.7
C3—C2—C9	118.4 (2)	C11—C10—C1	120.1 (3)
C3—C2—C1	117.9 (2)	C11—C10—H10	119.9
C9—C2—C1	123.7 (2)	C1—C10—H10	119.9
C4—C3—C2	121.0 (2)	C10—C11—C12	127.4 (3)
C4—C3—H3	119.5	C10—C11—H11	116.3
C2—C3—H3	119.5	C12—C11—H11	116.3
O2—C4—C3	118.0 (2)	C13—C12—C17	118.7 (2)
O2—C4—C7	122.1 (3)	C13—C12—C11	123.0 (2)
C3—C4—C7	119.9 (2)	C17—C12—C11	118.2 (2)
O2—C5—C6	110.0 (3)	C14—C13—C12	119.9 (3)
O2—C5—H5A	109.7	C14—C13—H13	120.0
C6—C5—H5A	109.7	C12—C13—H13	120.0
O2—C5—H5B	109.7	C15—C14—C13	119.4 (3)
C6—C5—H5B	109.7	C15—C14—H14	120.3
H5A—C5—H5B	108.2	C13—C14—H14	120.3
O3—C6—C5	111.2 (2)	C14—C15—C16	122.1 (3)
O3—C6—H6A	109.4	C14—C15—Cl1	118.6 (2)
C5—C6—H6A	109.4	C16—C15—Cl1	119.3 (2)
O3—C6—H6B	109.4	C15—C16—C17	118.5 (3)
C5—C6—H6B	109.4	C15—C16—H16	120.8
H6A—C6—H6B	108.0	C17—C16—H16	120.8
O3—C7—C8	118.5 (3)	C16—C17—C12	121.5 (3)
O3—C7—C4	122.0 (3)	C16—C17—H17	119.3
C8—C7—C4	119.5 (3)	C12—C17—H17	119.3
C7—C8—C9	120.5 (2)		
			
O1—C1—C2—C3	−8.9 (4)	C4—C7—C8—C9	1.3 (4)
C10—C1—C2—C3	169.9 (2)	C7—C8—C9—C2	−0.8 (4)
O1—C1—C2—C9	169.5 (3)	C3—C2—C9—C8	−0.2 (4)
C10—C1—C2—C9	−11.7 (4)	C1—C2—C9—C8	−178.6 (3)
C9—C2—C3—C4	0.7 (4)	O1—C1—C10—C11	7.4 (4)
C1—C2—C3—C4	179.1 (2)	C2—C1—C10—C11	−171.3 (3)
C5—O2—C4—C3	158.0 (3)	C1—C10—C11—C12	−179.0 (2)
C5—O2—C4—C7	−21.8 (4)	C10—C11—C12—C13	6.8 (4)
C2—C3—C4—O2	−180.0 (2)	C10—C11—C12—C17	−176.7 (3)
C2—C3—C4—C7	−0.1 (4)	C17—C12—C13—C14	−1.2 (4)
C4—O2—C5—C6	50.4 (3)	C11—C12—C13—C14	175.3 (2)
C7—O3—C6—C5	38.6 (4)	C12—C13—C14—C15	0.4 (4)
O2—C5—C6—O3	−60.1 (4)	C13—C14—C15—C16	0.9 (4)
C6—O3—C7—C8	171.8 (3)	C13—C14—C15—Cl1	−179.0 (2)
C6—O3—C7—C4	−9.4 (4)	C14—C15—C16—C17	−1.3 (4)
O2—C4—C7—O3	0.2 (4)	Cl1—C15—C16—C17	178.7 (2)
C3—C4—C7—O3	−179.6 (3)	C15—C16—C17—C12	0.4 (4)
O2—C4—C7—C8	179.0 (2)	C13—C12—C17—C16	0.8 (4)
C3—C4—C7—C8	−0.8 (4)	C11—C12—C17—C16	−175.8 (2)
O3—C7—C8—C9	−179.9 (3)		

### Hydrogen-bond geometry (Å, º)

**Table:**

*D*—H···*A*	*D*—H	H···*A*	*D*···*A*	*D*—H···*A*
C5—H5*A*···O3^i^	0.99	2.61	3.595 (4)	172
C5—H5*B*···Cl1^ii^	0.99	2.80	3.455 (3)	124

Symmetry codes: (i) *x*−1/2, −*y*+3/2, *z*; (ii) *x*−1, *y*, *z*−1.
